# Learning Adaptive Filtering in the Fourier Domain for Fringe Pattern Analysis: A New Reconstruction Paradigm in Digital Holographic Interferometry

**DOI:** 10.1002/nap2.70282

**Published:** 2026-10-16

**Authors:** Youxing Li, Lingzhi Meng, Zhiyuan Xu, Hongwei Li, Kai Zhang, Yaoqing Xie, Libo Yuan

**Affiliations:** ^1^ Key Lab of In‐Fiber Integrated Optics Ministry of Education of China Harbin Engineering University Harbin China; ^2^ Zhejiang Huadong Optic‐Electronic Instrument Co., Ltd. Jiaxing Zhejiang China; ^3^ Photonics Research Center, School of Optoelectronic Engineering Guilin University of Electronic Technology Guilin China

**Keywords:** deep learning, digital holographic interferometry, Fourier‐domain filtering, fringe pattern analysis

## Abstract

Single‐frame fringe pattern analysis in digital holographic interferometry (DHI) is essential for precision phase measurement under dynamic conditions. Recent advances in deep learning within optical metrology are transforming the reconstruction paradigm from physics‐based modeling toward data‐driven learning. However, most existing neural networks either rely on learning statistical visual features without guidance from physical principles or fail to dynamically adjust inference parameters based on the input instance after training, thereby limiting the robustness and generalization capacity on unseen data. To this end, a novel adaptive spectrum filtering neural network (ASFNet) is proposed for phase measurement. ASFNet incorporates a physics‐aware spectral filtering block that dynamically adapts the inference process to each input instance, enabling robust and generalizable phase reconstruction under varying and unseen conditions—moving beyond static, dataset‐specific statistical modeling toward instance‐aware adaptive reconstruction. Moreover, a forward model is constructed to generate realistic simulation data for network training, reducing the reliance on experimentally labeled data. Simulation results on 400 test samples demonstrate the superiority of ASFNet in terms of both pixel‐wise accuracy and visual quality. Experimental results further confirm its strong generalization capability on unseen data, achieving high reconstruction fidelity comparable to that of hand‐tuned, physics‐based methods.

## Graphical abstract

The object beam and the reference beam interfere at the camera sensor surface and multi‐angle tomographic recording based on off‐axis digital holography.

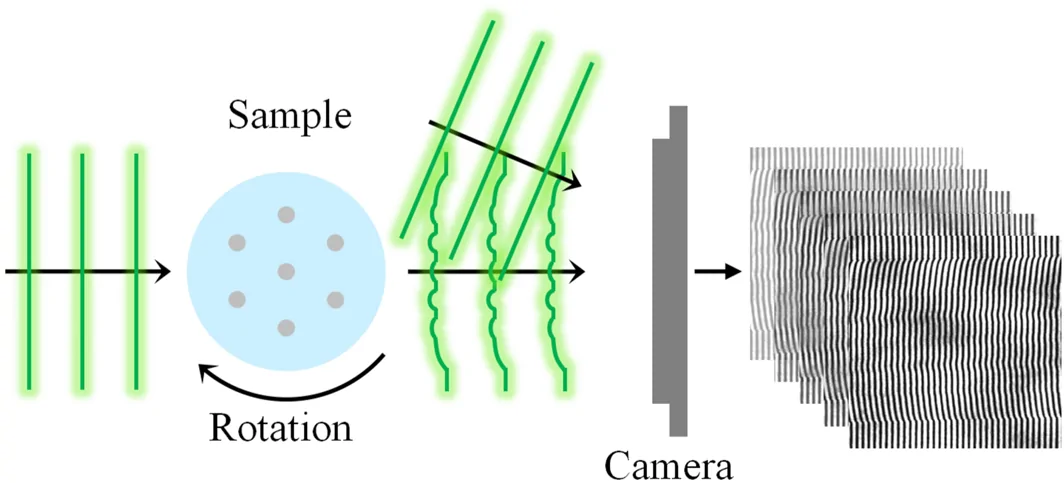

## 1 Introduction

Optical metrology enables noncontact and high‐precision measurement of physical parameters, such as surface topography, displacement, strain, and refractive index, by exploiting interference, diffraction, or modulation generated from light–object interactions. Leveraging its high sensitivity, full‐field measurement capability, and accurate perception of micro‐ and nanoscale structures, optical metrology has been widely applied in advanced manufacturing [1, 2], precision inspection [3, 4], biomedical imaging [5], and materials science [6]. Among existing optical metrology techniques, digital holographic interferometry (DHI) exhibits unique advantages for the quantitative imaging and measurement of semi‐transparent samples [7, 8, 9]. The measurement accuracy of DHI depends not only on the fidelity of the recording process but also on the reliability of fringe pattern analysis techniques during reconstruction, which requires stable and accurate phase demodulation from recorded fringe patterns. As a core inversion procedure in DHI, fringe pattern analysis is also essential for other optical metrology techniques, such as electronic speckle pattern interferometry [10] and fringe projection profilometry [11].

Traditional techniques mainly consist of phase‐shifting (PS) methods and spatial phase demodulation (SPD) methods. PS methods achieve pixel‐level high‐precision reconstruction from multiple fringe patterns but require high environmental stability during recording. SPD methods enable single‐frame fringe pattern analysis, offering advantages in measurement efficiency, resistance to environmental disturbances, and adaptability to dynamic scenes, making them particularly suitable for fast measurements in transient processes or unstable experimental conditions. Typical SPD methods mainly include Fourier transform analysis (FTA) [12], windowed Fourier transform [13, 14, 15], wavelet analysis [16, 17], and Hilbert transform‐based strategies [18]. These methods are primarily built upon well‐defined inversion principles and prior knowledge, achieving accurate measurement on clean fringe patterns. However, they rely on handcrafted analysis parameters to tune the inversion process, such as the shape of frequency‐domain filtering windows or local analysis scales. When fringe patterns are severely degraded by noise, defocus, or overexposure, it becomes difficult to determine appropriate parameter settings to ensure measurement accuracy. Consequently, achieving accurate phase demodulation from single‐frame degraded fringe patterns while reducing reliance on handcrafted design remains a significant challenge.

In recent years, deep learning has provided new solutions for solving complex inverse problems in optical metrology [19, 20, 21]. Unlike traditional methods, deep learning‐based approaches achieve high‐fidelity reconstruction in a data‐driven manner by leveraging neural networks. For fringe pattern analysis, various deep learning methods have been proposed. Feng et al. designed a framework cascading two convolutional neural networks (CNNs) for fringe pattern analysis, where CNN1 estimates the background intensity from the fringe pattern and CNN2 predicts the numerator and denominator terms to achieve accurate phase demodulation [22]. To improve measurement efficiency, Sun et al. proposed RU‐Net, which uses ResUNet as the backbone to simultaneously output the wrapped phase and the corresponding wrap count from a single interferogram, thereby accomplishing joint phase extraction and unwrapping [23]. Another common strategy is to employ a standalone neural network to directly predict the target physical quantities from fringe patterns [24, 25, 26, 27], demonstrating the powerful capability of deep learning for addressing ill‐posed inverse problems. Considering the limited representational capacity of a single network, Feng et al. proposed an ensemble strategy that integrates predictions from multiple baseline networks with different architectures to improve measurement stability [28]. These methods establish mappings between input fringe patterns and target variables through hierarchical feature extraction and spatial transformation performed by neural networks. They focus primarily on capturing statistical visual characteristics from the training data while rarely incorporating relevant physical principles or measurement knowledge. Consequently, when the test data exhibit style differences from the training samples, the generalization performance of the network may be compromised, leading to unpredictable errors.

To improve the reliability and robustness of intelligent measurement, physics‐informed neural networks (PINNs) have become increasingly popular. By embedding spectral analysis [29], integrating numerical propagation models [30], or imposing imaging constraints as loss functions [31], these methods can yield predictions that conform to statistical physical principles. However, these PINNs are unable to dynamically adjust the inversion process according to input instances because their inference weights are fixed after training. This characteristic limits both the applicability and the reconstruction accuracy of the method. Furthermore, the fringe patterns acquired under different scenarios exhibit substantial variations, such as fringe density, degradation level, and the characteristics of the target under measurement. These variations inevitably affect the process of phase demodulation. Therefore, enabling the network to perform physics‐aware adaptive transformations on features according to different inputs is expected to further enhance generalization on unseen scenarios.

For this purpose, a novel adaptive spectrum filtering neural network (ASFNet) is developed for fringe pattern analysis in DHI. ASFNet introduces a Fourier‐domain attention mechanism that enables the network to adaptively select object spectrum content after transforming the interferogram into features. By dynamically adjusting the spectral filtering weights, ASFNet can achieve stable and intelligent prediction on unseen samples. The proposed ASFNet achieves significantly higher accuracy on simulated data compared with the classical FTA. More importantly, ASFNet, trained exclusively on simulated data, maintains stable reconstruction fidelity on unseen experimental data, producing fewer outliers and artifacts than those obtained with a carefully handcrafted FTA. In this way, ASFNet moves beyond purely dataset‐specific statistical fitting toward instance‐aware adaptive reconstruction, offering an effective solution for intelligent fringe pattern analysis and optical metrology.

## 2 Methodology

### 2.1 Physics Principle in DHI

The object beam and the reference beam interfere at the camera sensor surface, as shown in Figure 1a, and the recorded interferometric pattern in DHI can be expressed as

(1) $$I\left(x,y\right)=I_{b}\left(x,y\right)+I_{m}\left(x,y\right)\cos\left[\phi \left(x,y\right)+2\pi f_{0}x\right],$$

where $I_{b}\left(x,y\right)$ represents the background intensity, $I_{m}\left(x,y\right)$ denotes the modulation amplitude, ϕ(x,y) is the modulated phase in the interferometry, and $2\pi f_{0}x$ represents the spatial carrier frequency term. According to the FTA method [12], the interferometric spectrum can be expressed as

(2) $$\tilde{I}\left(f_{x},f_{y}\right)=\tilde{I_{b}}\left(f_{x},f_{y}\right)+\tilde{C}\left(f_{x},f_{y}\right)+\tilde{C^{*}}\left(f_{x},f_{y}\right),$$

with

(3) $$C\left(x,y\right)=\frac{1}{2}I_{m}\left(x,y\right)\exp\left\{i\left[\phi \left(x,y\right)+2\pi f_{0}x\right]\right\},$$

where $\tilde{I_{b}}$ represents the spectrum of the reference beam autocorrelation, also referred to as the zero‐order spectrum. $\tilde{C}$ denotes the spectrum associated with the object beam (also termed the +1st‐order spectrum). $\tilde{C^{*}}$ is the conjugate of $\tilde{C}$, corresponding to the −1st‐order spectrum. In off‐axis digital holography, the +1st‐order spectrum does not overlap with the zero‐order and −1st‐order spectra, so it can be extracted through digital filtering:

(4) $$\tilde{C}\left(f_{x},f_{y}\right)=\tilde{I}\left(f_{x},f_{y}\right)⊙{\tilde{w}}_{1}\left(f_{x},f_{y}\right)⊙{\tilde{w}}_{2}\left(f_{x},f_{y}\right).$$

**FIGURE 1:**
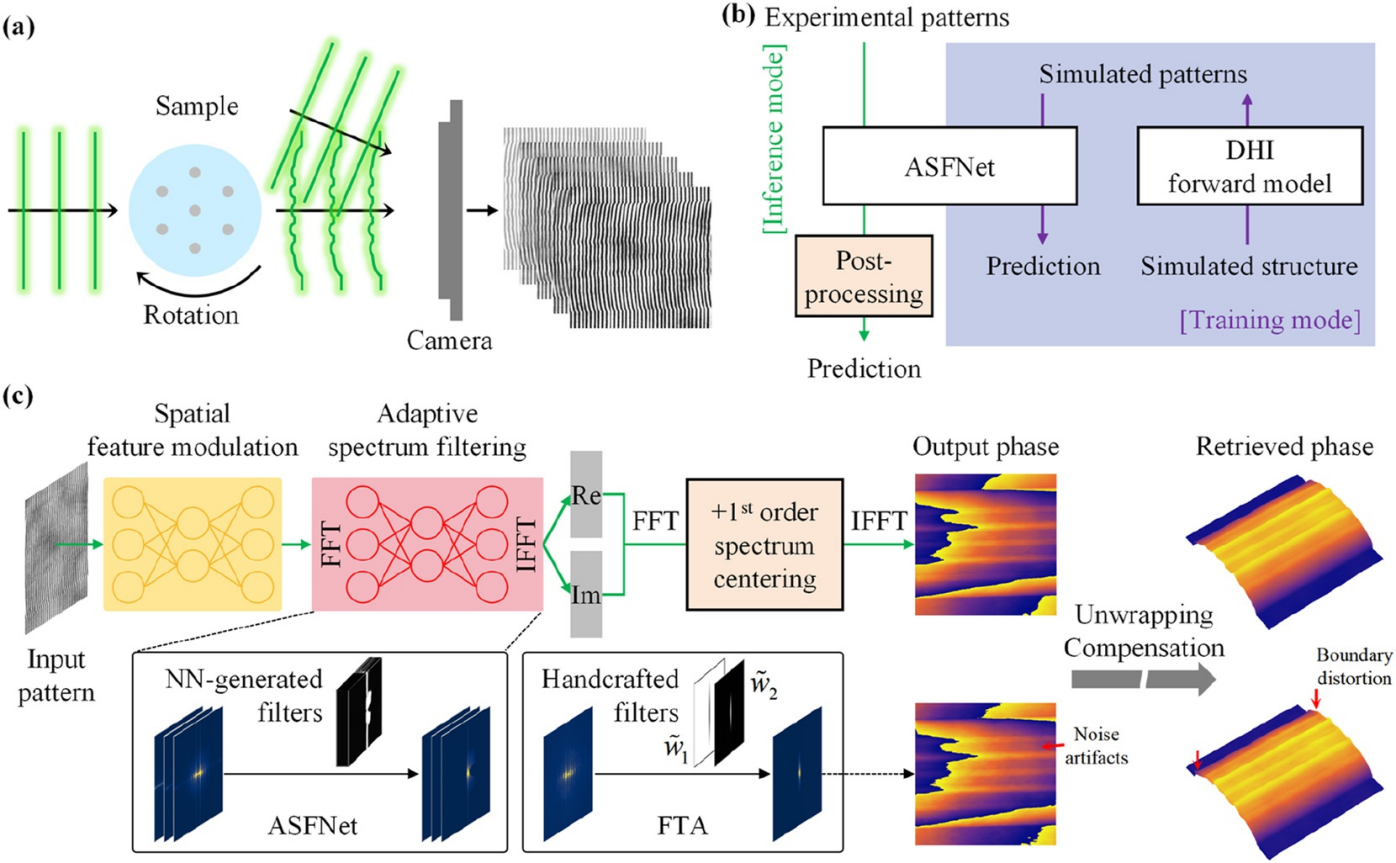
Schematic representation of DHI and the phase demodulation workflow of ASFNet. (a) Multiangle tomographic recording based on off‐axis digital holography. (b) Training and inference pipeline of ASFNet. (c) Phase demodulation of ASFNet in inference mode, comprising spatial‐domain feature modulation, Fourier‐domain adaptive filtering, and postprocessing to center the +1st‐order spectrum.

Subsequently, the filtered components are shifted to the spectrum center to remove the linear phase introduced by the carrier frequency:

(5) $$\tilde{U}\left(f_{x},f_{y}\right)=\tilde{C}\left(f_{x}+f_{0},f_{y}\right).$$

Then, the phase distribution can be calculated as follows:

(6) $$\varphi \left(x,y\right)=\arctan\left\{\frac{\text{Im}\left[U\left(x,y\right)\right]}{\text{Re}\left[U\left(x,y\right)\right]}\right\},$$

where Im[U(x,y)] and Re[U(x,y)] represent the imaginary and real parts of a complex amplitude distribution. The demodulated phase is confined to the range (−π, π]. Therefore, phase unwrapping is required to eliminate 2π discontinuities [32], followed by aberration compensation to remove inherent optical system phase distortions [33], thereby recovering the true phase of the measured sample.

However, FTA requires a manual selection of appropriate filters for isolating the +1st‐order spectrum. When the measured phase exhibits significant variations, the object spectrum may broaden and overlap with other spectral components, making spectral filtering difficult. Furthermore, for fringe patterns with low signal‐to‐noise ratio, the FTA struggles to simultaneously achieve noise suppression and preservation of edge details, hindering high‐precision measurement.

### 2.2 Adaptive Spectrum Filtering Neural Network (ASFNet)

ASFNet is trained using a supervised learning strategy. As illustrated in Figure 1b, to construct the paired training data, degraded fringe patterns and corresponding phase ground‐truth (GT) are first synthesized using simulated fibers based on the DHI forward model. During training, the network takes simulated patterns as input and the wrapped phase containing the carrier term as GT, optimizing its parameters by minimizing the loss between its predicted phase and GT. During inference, the network reconstructs the pseudo‐complex amplitude distribution from the input and subsequently removes the carrier term to compute the wrapped phase, as shown in Figure 1c. ASFNet first performs feature extraction and spatial modulation on the input pattern, followed by adaptive spectral filtering to extract the +1st‐order spectrum. The filtered Fourier‐domain features are then transformed back to the spatial domain, where convolutional layers regress the real and imaginary parts of the complex distribution to compute the wrapped phase, achieving stable reconstruction.

The architecture of ASFNet in training mode is illustrated in Figure 2a. As shown, the network first extracts initial features through convolutional layers and then captures contextual information from these features via multiple residual dense blocks (RDBs), thereby achieving spatial‐domain feature modulation. As shown in Figure 2b, RDB expands the receptive field by stacking multiple feature transform layers (FTLs) and concatenates features at different levels through skip connections. Subsequently, feature fusion is performed via convolution, followed by residual addition with the input features to enhance the network representation capacity and improve training stability. Figure 2c depicts the structure of FTL, which constructs multiscale representations by down‐sampling the input features at different factors and extracts contextual information through convolutional layers. After being restored to their original resolution via up‐sampling, features at each scale are concatenated along the channel dimension and fused through a convolutional layer, followed by residual addition with the input to complete the feature transformation. Afterward, the network converts the features to the Fourier domain for adaptive spectral filtering. As shown in Figure 2d, an adaptive spectrum filtering block (ASFB) is designed to explicitly perform object spectrum selection. ASFB first fuses the real and imaginary parts of the input spectral features through a convolutional layer and then uses RDBs to further enhance contextual representation. Subsequently, a convolutional layer followed by a Sigmoid function generates spectral attention weights ranging in [0, 1], aiming to enhance the +1st‐order components while suppressing irrelevant ones. From the perspective of spectral analysis, the structure of ASFB makes the feature‐inference process formally consistent with the Fourier‐domain filtering procedure in FTA. Therefore, ASFB can be regarded as a learnable and instance‐adaptive counterpart of the spectral filtering step in FTA. To further verify this interpretation, we visualized and discussed the intermediate features and attention maps inside ASFB, with detailed visualizations provided in Supporting Information S1.

**FIGURE 2:**
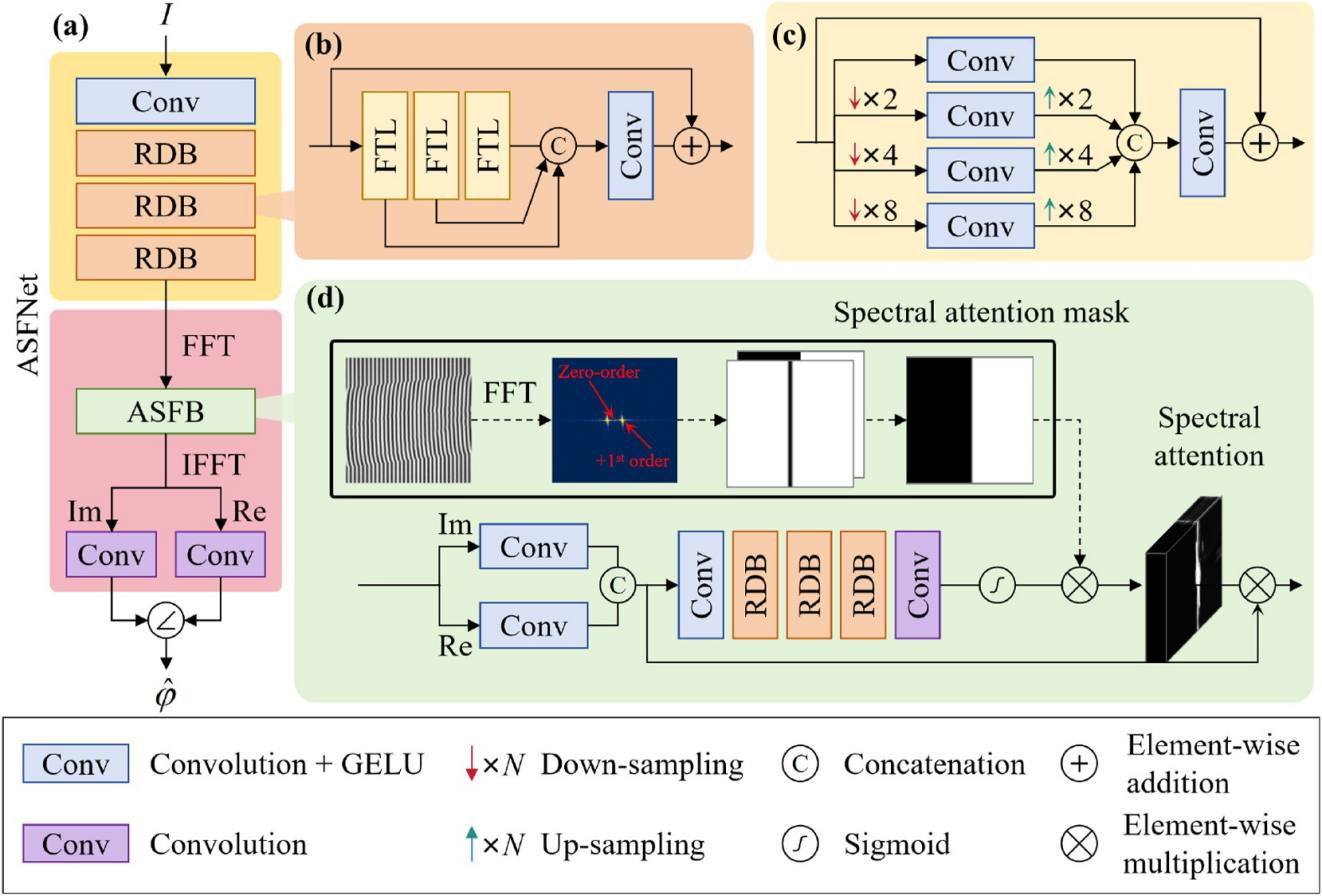
Architecture of ASFNet and its components. (a) ASFNet in training mode. (b) RDB for capturing rich contextual information. (c) FTL is the core component of each RDB that enables multiscale feature extraction. (d) ASFB for adaptive Fourier‐domain filtering, where the spectrum attention mask provides a coarse constraint on the candidate +1st‐order spectral region and the generated spectral attention weights further perform instance‐specific filtering.

To improve training stability, we also introduce a spectrum attention mask (SM) to provide a coarse physics‐informed constraint for the ASFB. According to the Fourier spectral structure of off‐axis fringe patterns, the zero‐order spectrum is mainly distributed around the central region, whereas the conjugate spectrum is located on the opposite side of the object spectrum. Therefore, the SM is constructed by combining two masks: one suppresses the central zero‐order spectral region using an inverted Hanning window, whereas the other suppresses the half‐plane corresponding to the conjugate spectrum. The SM is obtained by multiplying these two masks and is then multiplied with the attention weights, thereby effectively constraining the automatic search range of the ASFB for the object spectral components and reducing unstable responses caused by irrelevant spectral components. The filtered features are then transformed back to the spatial domain, yielding complex feature representations. Finally, convolutional layers are used to reconstruct the real and imaginary parts of the pseudo‐complex amplitude, from which the wrapped phase is retrieved via the arctan function.

We adopt the mean absolute error loss function to optimize ASFNet:

(7) $$\text{Loss}={\left\|\hat{\varphi }-\varphi_{\text{GT}}\right\|}_{1},$$

where $\hat{\varphi }$ denotes the network prediction and $\varphi_{\text{GT}}$ denotes the GT.

## 3 Experiments and Results

### 3.1 Data Preparation

We established a forward model for off‐axis digital holographic microtomography, which generates interferograms and phase labels by simulating the three‐dimensional refractive index (3D RI) distribution of fibers. As shown in Figure 3a, to better approximate the appearance of experimentally acquired fringe patterns, we added noise and overexposure effects to clean patterns. More details of the simulation procedure are provided in Supporting Information S1. According to the simulation pipeline, a total of 4400 paired data samples were generated and randomly split into training and validation sets in a 10:1 ratio.

**FIGURE 3:**
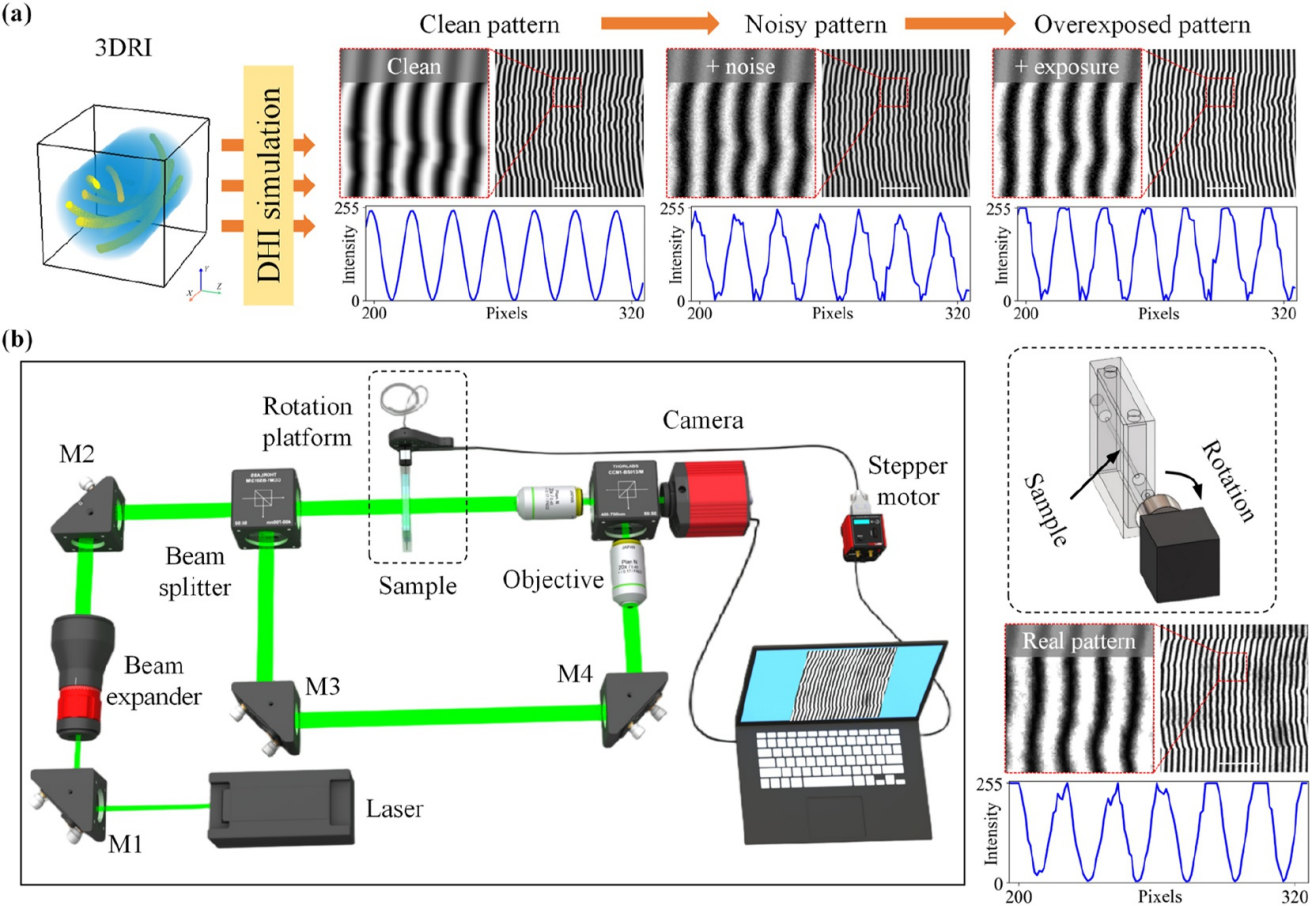
Simulated and experimental data. (a) Simulation of DHI on fibers. (b) Schematic representation of the digital holographic microtomography setup, with a recorded pattern on the right.

A digital holographic microtomography system was further configured to record real‐world interferograms. As illustrated in Figure 3b, a 532‐nm laser serves as the light source. The beam is expanded and then split into object and reference arms. The object beam illuminates the sample and is magnified by a bright‐field objective (20×/0.40 NA), whereas the reference beam propagates along an independent path. They recombine at the prism, generating spatially modulated interference patterns at the camera sensor plane, with a spatial resolution of 1600 × 1200 pixels and a pixel size of 3.45 μm × 3.45 μm. The fiber is mounted on a rotation stage driven by a stepper motor for equiangular stepwise rotation, whereas the camera synchronously records interferograms. The acquired patterns are then cropped to 512 × 512 pixels for testing.

### 3.2 Implementation Details

The network was optimized using the Adam optimizer [34] with a total of 100 training epochs. The initial learning rate was set to 1 × 10^−3^ and progressively reduced to 1 × 10^−6^ following a cosine annealing schedule [35]. Data augmentation was performed via random horizontal flipping during training. The network was implemented in Python 3.8 with PyTorch 1.12.1 and trained on a server equipped with two RTX 3090 GPUs.

### 3.3 Validation on Simulated Patterns

We first evaluated the proposed method on simulated data. Because the performance of FTA is highly dependent on manually selected spectral filters, it is necessary to determine its optimal configuration before performing a fair comparison with deep learning‐based methods. To this end, we configured four FTA schemes using Hanning windows of different scales (denoted as win1–win4) and tested them on two representative interferograms with different fringe periods and degradation effects, as shown in Figure 4a. The defined Hanning windows are illustrated in Figure 4b. A peak search operation was performed on the input spectrum to locate the zero‐order and +1st‐order spectral peaks. The vertical distance *d* between these two peak coordinates was then computed and used as the reference for window sizing. Subsequently, four windows were constructed centered at the +1st‐order spectral peak: win1 (height 4*d*, width 2*d*), win2 (8*d*, 2*d*), win3 (12*d*, 2*d*), and win4 (12*d*, 3*d*). Meanwhile, we visualized the spectral attention map generated by ASFNet, which is aggregated from the multichannel attention weights produced by ASFB. Specifically, each channel‐wise attention map was assigned a scalar coefficient calculated as the sum of the spatially averaged absolute responses of the real and imaginary parts of its corresponding complex feature spectrum. All weighted attention maps were then summed to obtain the final visualization map. The detailed construction procedure is provided in Supporting Information S1. Figure 4c presents the phase demodulation errors of different methods on the two representative cases. Among the four FTA configurations, FTA‐win4 achieved the best reconstruction accuracy and was therefore selected as the tuned FTA configuration for subsequent statistical comparisons. Nevertheless, ASFNet still achieved lower reconstruction errors than FTA‐win4 on both cases. Furthermore, we analyzed the dynamic perception accuracy of ASFB based on the spectra involved in phase demodulation and the generated filter weights under different noise levels and fringe densities, with detailed results provided in Supporting Information S1. The attention learned by ASFNet consistently focused on the vicinity of the +1st‐order spectral region and adaptively adjusted its weighting range according to fringe frequency and degradation level, which partly explains its superior robustness and reconstruction accuracy over handcrafted filtering windows in FTA.

**FIGURE 4:**
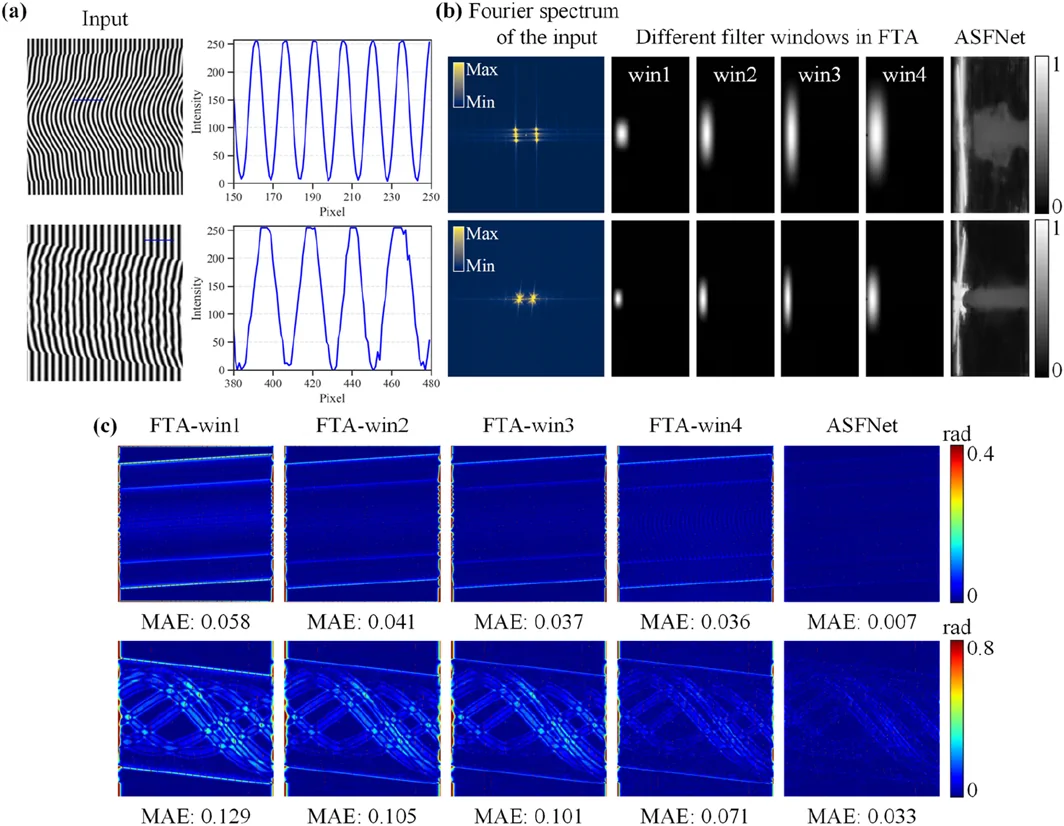
Phase demodulation results of FTA and ASFNet. (a) Interferograms with varying fringe periods, noise, and overexposure effects, along with intensity cross‐sections along the blue solid lines. (b) Fourier spectra of the interferograms in (a), Hanning windows with different scales configured in FTA, and the spectral attention maps in ASFNet. (c) Reconstruction accuracy of different methods.

For comprehensive evaluation, we further compared ASFNet (0.50 M parameters) with tuned FTA (FTA‐win4) and end‐to‐end ResUNet (1.28 M parameters). To ensure a fair comparison, ResUNet was trained with the same settings as ASFNet, including the Adam optimizer, learning‐rate schedule, number of training epochs, weight decay, and data augmentation strategy. The same L1 loss function was also adopted for ResUNet training. Quantitative evaluations were conducted on the validation set using four metrics: mean absolute error (MAE), root mean square error (RMSE), peak signal‐to‐noise ratio (PSNR), and structural similarity index measure (SSIM). For each metric, we report the mean and standard deviation (STD). Metric definitions are provided in Supporting Information S1. As shown in Figure 5a, ASFNet achieves the best performance across all four metrics, with the lowest MAE and RMSE, the highest PSNR and SSIM, and the smallest STD among all methods. Specifically, compared with the suboptimal ResUNet, ASFNet reduces MAE and RMSE by approximately 39% and 10.6%, respectively. In contrast, FTA yields lower performance with the largest STD. Figure 5b presents a qualitative comparison of the demodulated phase by different methods. As shown, ASFNet demonstrates the best measurement accuracy. The prediction errors of ResUNet are mainly concentrated in regions with dense edges, whereas the errors by FTA are distributed across the entire field of view. To intuitively visualize the measurement differences, the demodulated phases were further processed by spatial phase unwrapping and aberration compensation to reconstruct the fiber projection phase, as shown in Figure 5c. It can be seen that FTA tends to oversmooth the fiber core details. The results provided by ResUNet exhibit obvious spike artifacts, which severely degrade fidelity and cause a low SSIM. By contrast, ASFNet achieves high‐fidelity reconstruction, with its results maintaining a higher similarity to the GT and exhibiting the fewest artifacts. Figure 5d shows the phase cross‐sectional profiles along the dashed lines L1 and L2 in Figure 5c. The phase curve predicted by ASFNet closely matches the GT, validating its advantage in measurement accuracy.

**FIGURE 5:**
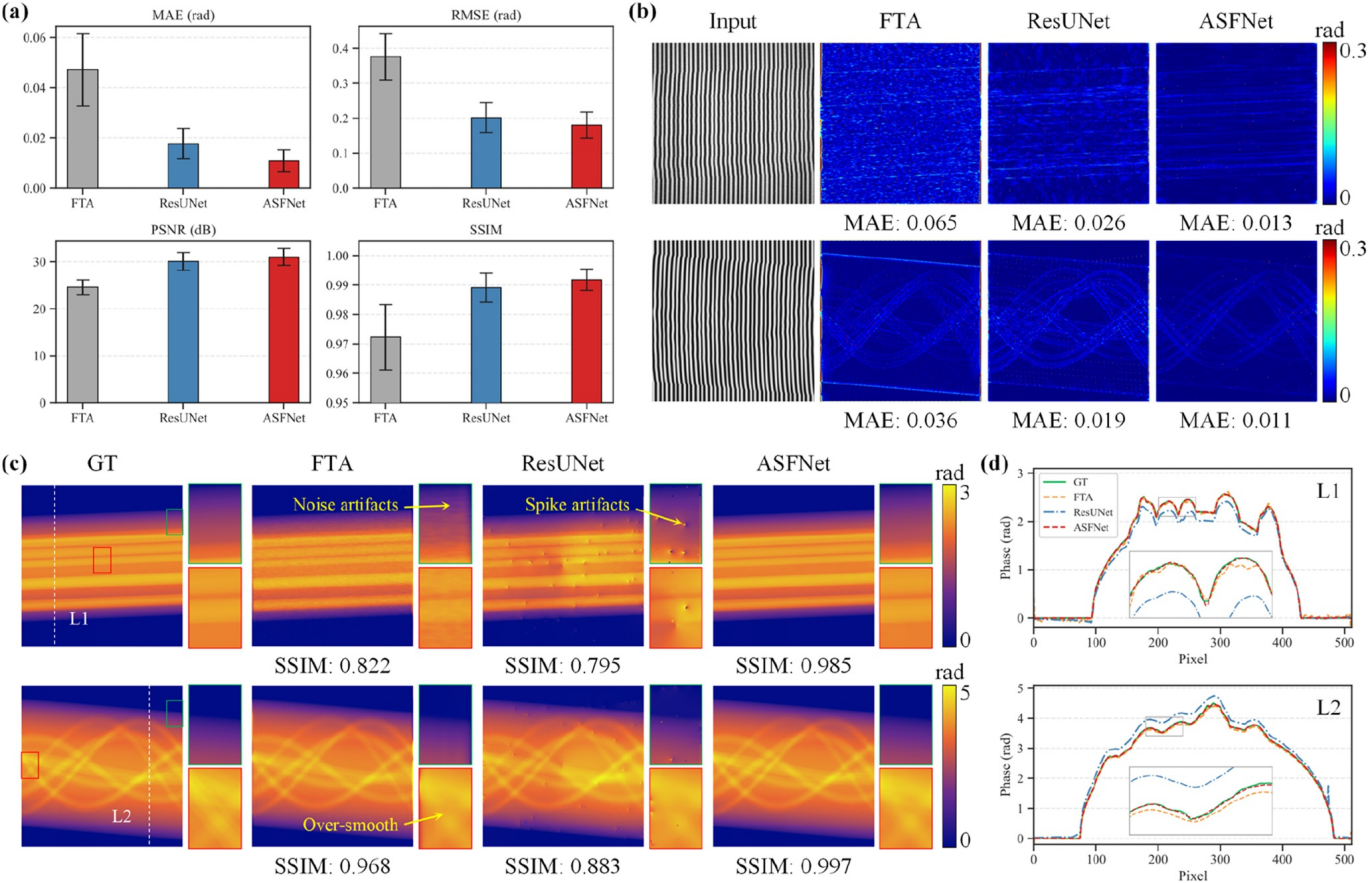
Comparison of simulation results by different methods. (a) The mean and STD of MAE, RMSE, PSNR, and SSIM over 400 simulation cases. (b) Example of a degraded interferogram and the phase demodulation error by FTA, ResUNet, and ASFNet. (c) Reconstructed object projection phase. (d) Phase profiles along the white dashed lines L1 and L2 in (c).

To further evaluate the practical deployment potential of ASFNet, we compared the computational performance of FTA, ResUNet, and ASFNet on a 512 × 512 single‐frame interferogram. As shown in Table 1, FTA achieves the shortest inference time. ResUNet has lower computational complexity and faster inference than ASFNet; however, as demonstrated earlier, its reconstruction accuracy and stability are clearly inferior. ASFNet incurs a higher computational cost, mainly because of the Fourier‐domain complex feature transformation and adaptive spectral filtering operations. Nevertheless, ASFNet achieves an inference time of 0.0289 s per frame, corresponding to approximately 34 frames per second, indicating its potential for online DHI phase reconstruction. In addition, ASFNet has fewer trainable parameters than ResUNet, suggesting that its improved performance is not simply achieved by increasing the model size.

**TABLE 1:** Computational performance comparison of different methods for a 512 × 512 single‐frame interferogram.

Methods	Inference time (s)	GFLOPs	Inference GPU memory (MiB)	Parameters
FTA	0.0026 s	—	—	—
ResUNet	0.0038 s	15.96	1884 MiB	1.28 M
ASFNet	0.0289 s	104.44	2260 MiB	0.50 M

### 3.4 Ablation Study on the ASFNet Architecture

To validate the effectiveness of each component within ASFNet, we conducted a series of ablation studies on spatial feature modulation (SFM), ASFB, and SM within the ASFB. As shown in Figure 6a, the demodulation accuracy of the network without SFM decreases significantly. Furthermore, removing the ASFB (w/o ASFB) also leads to a degradation in network performance, although its impact is less pronounced compared with the removal of SFM. These results indicate that both SFM and ASFB play crucial roles in enhancing measurement performance. Furthermore, we evaluated the necessity of the SM within the ASFB. Figure 6b illustrates the loss function during training and the RMSE variation curve on the validation set. Without SM, the loss error increases substantially in the early stage of training, accompanied by a marked reduction in stability. We repeated the training multiple times under the same configuration and consistently observed divergence after approximately 10 epochs. This demonstrates that incorporating SM in ASFB is essential for improving network stability.

**FIGURE 6:**
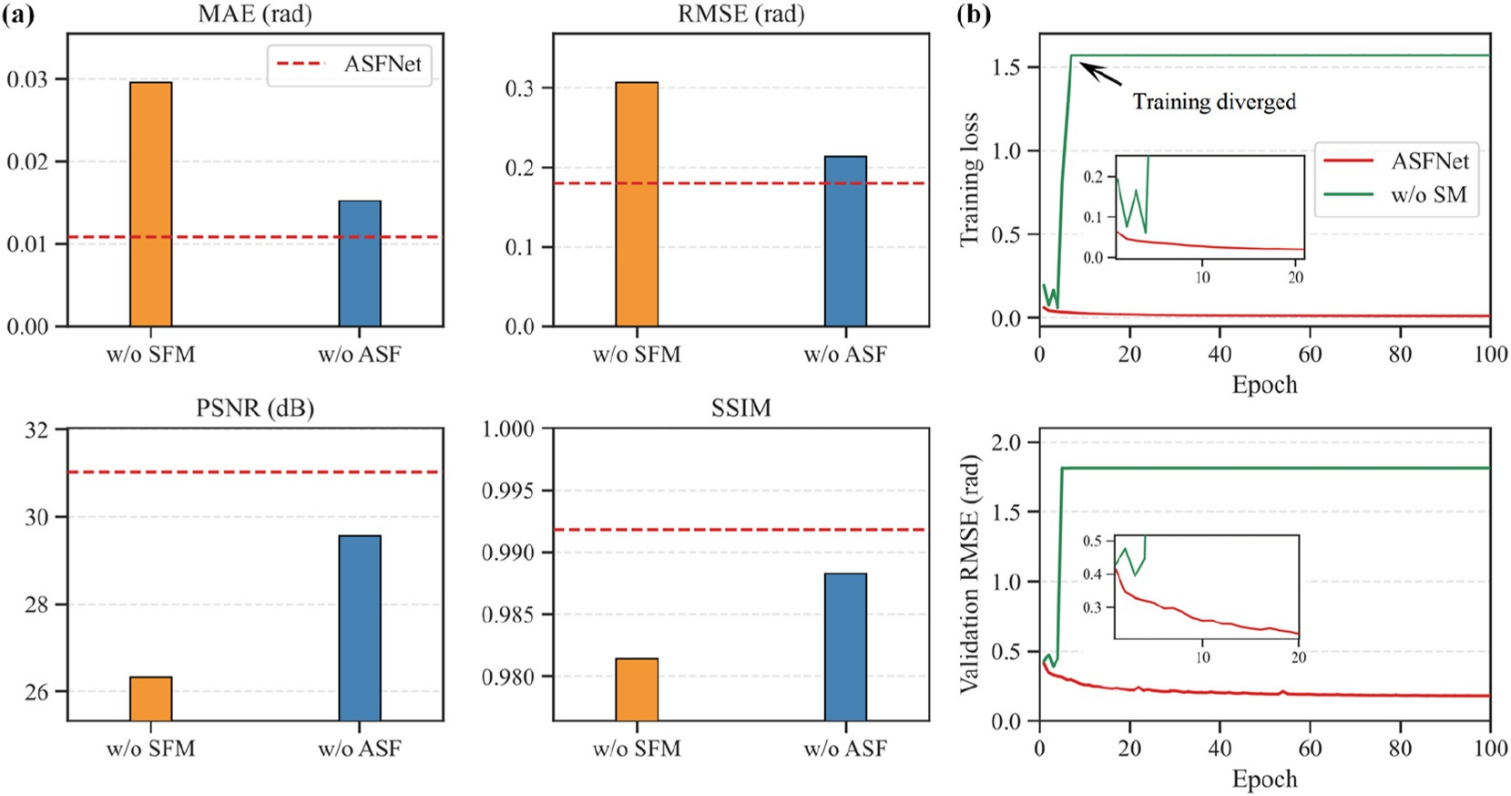
Ablation results of ASFNet. (a) Performance comparison of the network before and after removing SFM or ASFB. (b) Training loss and RMSE curves on the validation set with and without the SM.

### 3.5 Generalization on Experimental Interferograms

Precise GT is unavailable for experimental interferograms, making quantitative evaluation infeasible. As an alternative, we first applied FTA with Hanning windows of different sizes (win1–win4) to the experimental data and selected the visually optimal reconstruction as a reference benchmark. The effectiveness of ASFNet was then qualitatively assessed by comparing its results with those obtained from the optimally configured FTA. Figure 7a presents the recorded interferogram and its intensity profile along the blue solid line. The interferogram exhibits pronounced brightness inhomogeneity, noise, and localized overexposure, causing the cross‐sectional intensity profile to deviate substantially from a sinusoidal waveform. Visually, FTA‐win1 yields an over‐smoothed projection phase with noticeable detail distortion in the fiber core region, whereas FTA‐win4 introduces prominent harmonic artifacts in the result. Although FTA‐win2 and FTA‐win3 show improvement over win1 and win4, their results still exhibit discernible boundary distortion, which is consistent with the cross‐sectional phase profiles in Figure 7d. By comparison, ASFNet produces visually superior reconstructions with fewer artifacts and outliers, demonstrating excellent stability.

**FIGURE 7:**
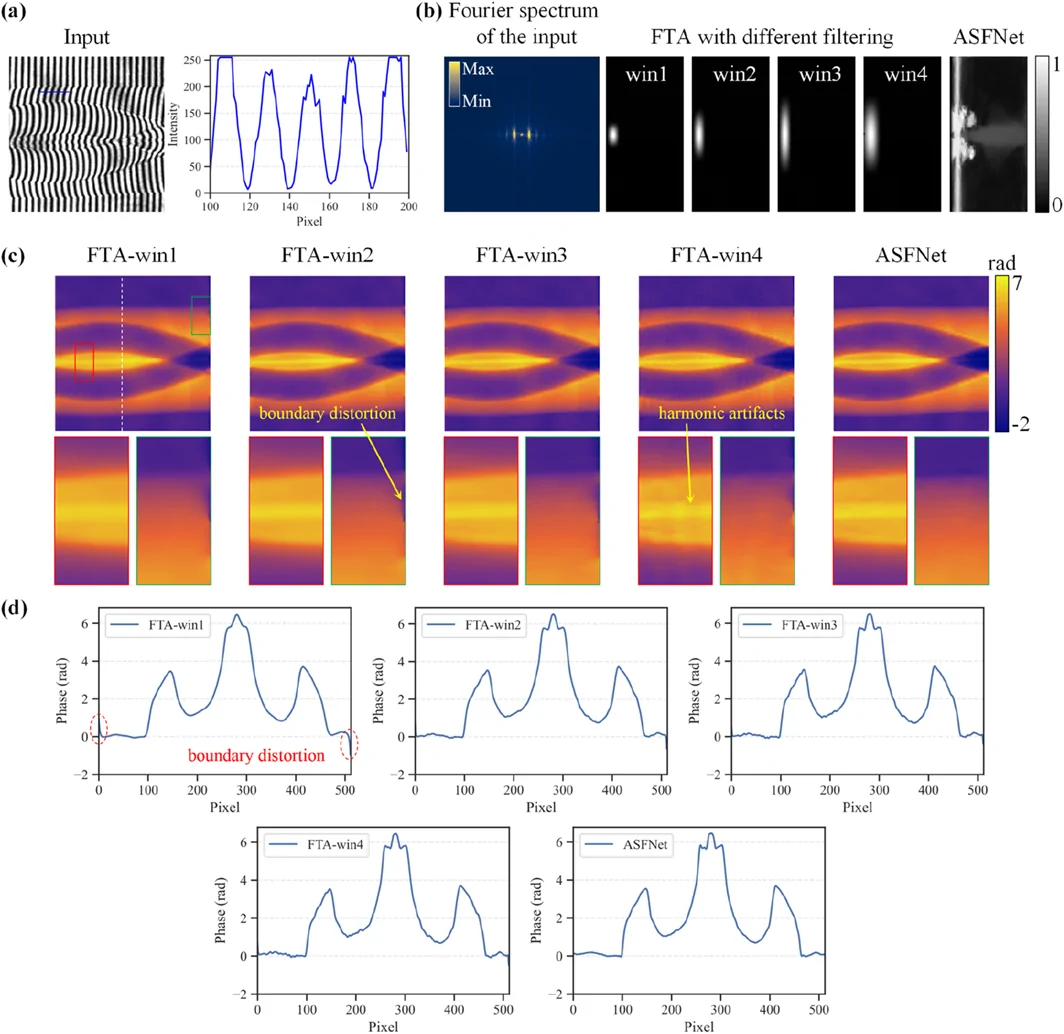
Experimental results provided by FTA and ASFNet. (a) Experimental interferogram and its distorted fringe. (b) Fourier spectrum of the interferogram, the Hanning windows with different scales configured in FTA, and the spectral attention map generated by ASFNet. (c) Projection phase provided by the different methods in (b). (d) The phase profiles along the white dashed lines in (c).

Figure 8a presents the interferograms captured under three different scenarios, along with the corresponding reconstructions obtained using FTA (optimally configured win3), ResUNet, and ASFNet. In Scenario 1, the fiber induces pronounced fringe deformation, leading to localized defocusing in the recorded interferogram. In these defocused regions, FTA suffers from unstable reconstructions and exhibits harmonic artifacts and edge distortion in the recovered projection, whereas ResUNet yields obvious spike artifacts. By contrast, ASFNet achieves stable reconstruction, although with slightly stripe‐like artifacts. In Scenario 2, the visual differences among the three methods remain largely consistent with Scenario 1. In Scenario 3, the fringe period becomes smaller. The distortions of FTA results are markedly reduced, whereas the prediction by ResUNet still suffers from severe artifacts. ASFNet delivers visually more pleasing reconstruction quality, with stripe‐like artifacts substantially suppressed in the result. Figure 8b presents the cross‐sectional phase profiles along the white dashed line in Figure 8a. Across all three scenarios, ASFNet predictions closely align with those from optimally configured FTA while exhibiting smaller local fluctuations, demonstrating strong generalization to unseen data. Moreover, ASFNet effectively suppresses the boundary distortion present in the FTA results and does not rely on handcrafted configuration, offering notable efficiency gains for phase demodulation in unknown scenarios. More results are provided in Supporting Information S1.

**FIGURE 8:**
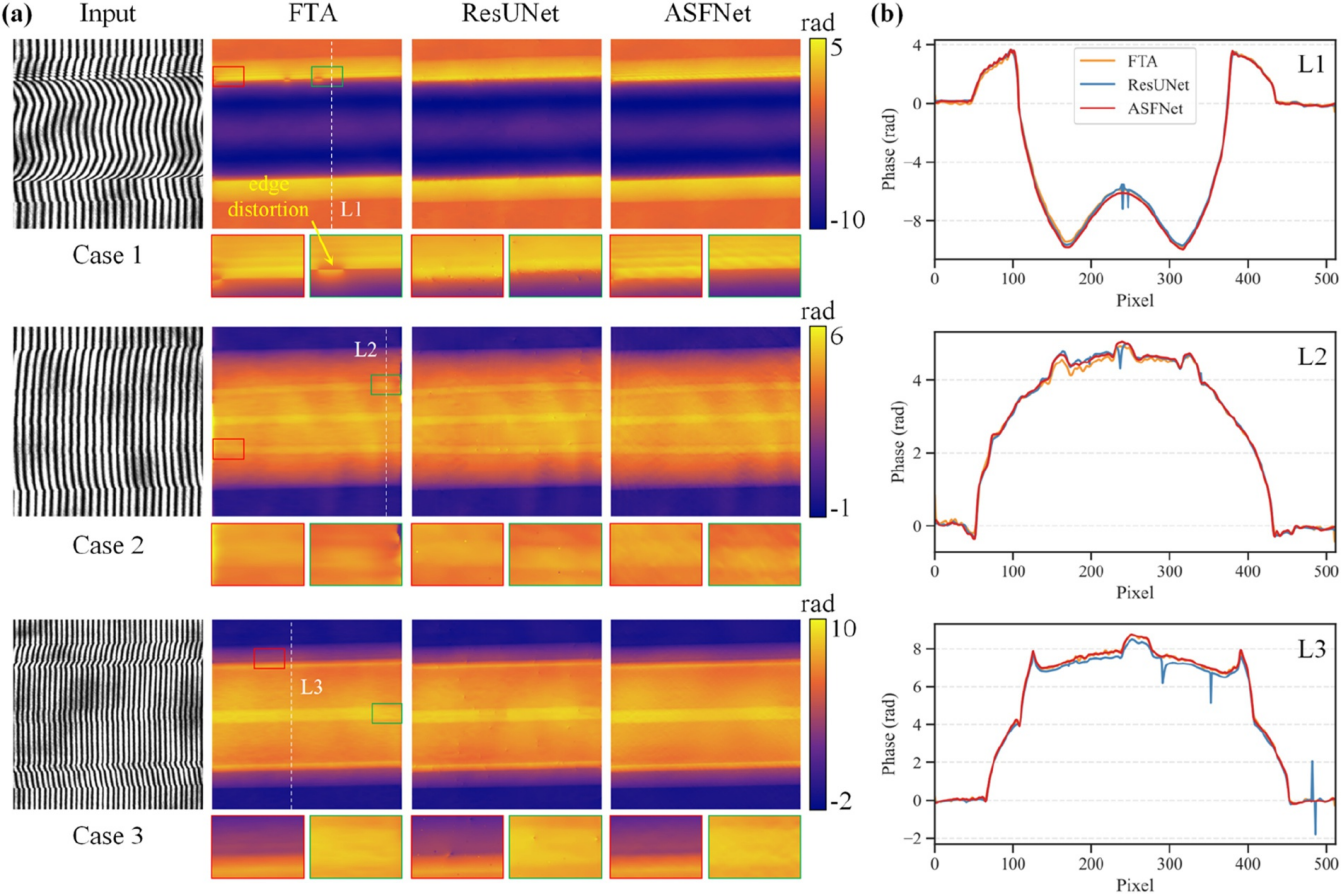
Experimental results obtained with FTA, ResUNet, and ASFNet. (a) Reconstructed object projection phase under different scenarios. (b) Cross‐sectional phase profiles along the white dashed lines in (a).

### 3.6 Application in Digital Holographic Tomography

Finally, we applied ASFNet to 3D RI reconstruction in digital holographic tomography (DHT). By controlling the rotation platform in the optical setup, the test fiber was rotated uniformly from 0° to 180° in 1° steps, and interferograms were recorded at each angular position. Phase demodulation was then performed on all 180 interferograms using FTA, ResUNet, and ASFNet to obtain wrapped phases, followed by unwrapping and compensation to yield multiangle projection phase maps. Figure 9a shows the images acquired at 0°, 15°, and 30°, together with the measured projection phase distributions. After obtaining all 180 projection phases, we performed position alignment and optical diffraction tomographic reconstruction to obtain the 3D RI distribution. Figure 9b presents the reconstructed RI at *Z* = 256 pixels. For reference, Figure 9c shows a microscopic image of the real fiber, with brighter regions indicating higher RI. As shown, the RI distribution reconstructed by FTA exhibits pronounced ripple artifacts near the outer cladding edge while suffering noticeable blurring at the cladding edges. The RI map obtained by ResUNet contains obvious scratch‐like artifacts, which are caused by spurious phase jumps in the recovered phase. In contrast, ASFNet achieves a well‐balanced reconstruction, with sharper boundaries, a cleaner background region, and the fewest visual artifacts. Figure 9d presents the cross‐sectional refractive index profiles along the white dashed line in Figure 9b. The RI profiles predicted by ASFNet exhibit smaller fluctuations within homogeneous regions and reveal more distinct contrasts among the surrounding medium, cladding, and core.

**FIGURE 9:**
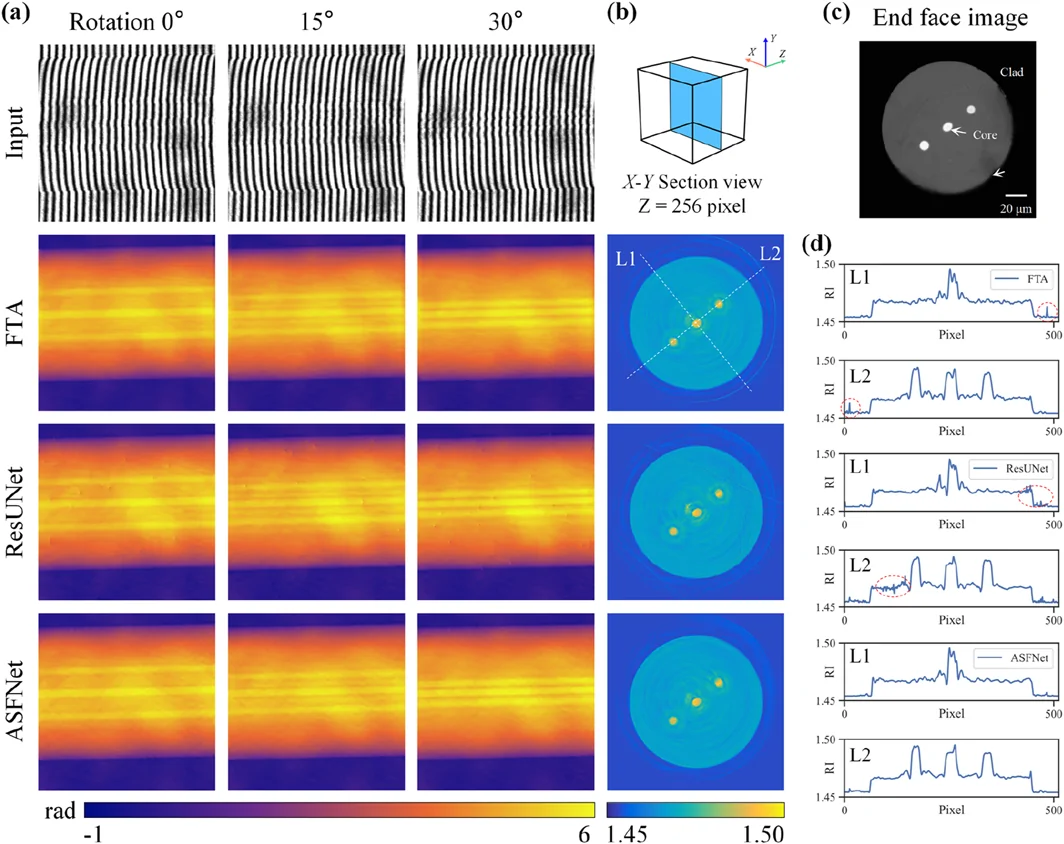
Experimental results of DHT. (a) Interferograms acquired at different rotation angles and the reconstructed projection phase. (b) RI distribution at the tomographic slice of *Z* = 256 pixels, reconstructed from 180 angular projections. (c) Microscopic image of the fiber end‐face captured under an optical microscope. (d) Cross‐sectional RI profiles along the white dashed line in (b).

## 4 Conclusion

In summary, this paper proposed an intelligent ASFNet for accurate phase reconstruction in DHI, which exhibits strong generalization capability on unseen data. ASFNet incorporated a physics‐aware adaptive spectral filtering block that dynamically adjusts its filtering strategy, ensuring stable and accurate measurements under different degraded scenarios. Simulation results demonstrate that ASFNet outperforms the classical FTA and end‐to‐end ResUNet across multiple quantitative metrics and in terms of visual reconstruction quality. Furthermore, ASFNet achieves stable reconstruction on unseen experimental data, outperforming well‐configured FTA in terms of visual quality across different scenarios, demonstrating its exceptional generalization capability.

It should be noted that although noise and overexposure effects were incorporated into the simulated data to better approximate experimental conditions, the domain gap between simulated and real‐world fringe patterns can hardly be eliminated. This gap mainly arises from the complex and coupled degradations present in real interferograms, including nonuniform illumination, environmental and system noise, defocus blur, local saturation, and mechanical vibration disturbances, which are difficult to fully reproduce using physical simulation models and thus limit the generalization performance of ASFNet on experimental data. To further reduce the inter‐domain discrepancy, several strategies can be considered in future work. First, the simulation pipeline can be improved by introducing domain randomization, allowing the network to encounter a wider range of fringe contrasts, carrier frequencies, noise levels, blur kernels, saturation conditions, and aberration patterns during training. Second, unlabeled experimental interferograms can be introduced as training data through self‐supervised or unsupervised learning [36, 37], although constructing reliable pseudo‐labels remains an open problem. Finally, domain adaptation techniques can be used to align the feature distributions between simulated and experimental data, thereby mitigating cross‐domain statistical discrepancies [38]. For example, image‐to‐image translation or style‐transfer strategies may be used to narrow the appearance difference between simulated and real fringe patterns while preserving the underlying phase information.

## Author Contributions

**Youxing Li:** conceptualization, methodology, software, data curation, investigation, writing – original draft. **Lingzhi Meng:** methodology, data curation, formal analysis. **Zhiyuan Xu:** data curation, investigation. **Hongwei Li:** software, data curation. **Kai Zhang:** formal analysis. **Yaoqing Xie:** data curation, formal analysis. **Libo Yuan:** supervision, methodology, funding acquisition, project administration, resources, writing – review and editing.

## Conflicts of Interest

The authors declare no conflicts of interest.

## Supporting information


Supporting Information S1

## Data Availability

The data that support the findings of this study are available from the corresponding author upon reasonable request.
