# Supplementary material for: Learning Adaptive Filtering in the Fourier Domain for Fringe Pattern Analysis: A New Reconstruction Paradigm in Digital Holographic Interferometry
Source: Nanophotonics. 2026 Oct 16;15(20):e70282. doi: 10.1002/nap2.70282 (PMC13675605; doi:10.1002/nap2.70282)
Supplement: Supplementary file 1 [file NAP2-15-e70282-s001.docx]

Supplementary Material

Learning adaptive filtering in the Fourier domain for fringe pattern analysis: A new reconstruction paradigm in digital holographic interferometry

Youxing Li^1^, Lingzhi Meng^2^, Zhiyuan Xu^1^, Hongwei Li^3^, Kai Zhang^1^, Yaoqing Xie^3^, and Libo Yuan^1, 3,^ *

^1^Key Lab of In-fiber Integrated Optics, Ministry Education of China, Harbin Engineering University, Harbin 150001, China

^2^Zhejiang Huadong Optic-Electronic Instrument Co., Ltd., Jiaxing 314001, Zhejiang, China

^3^Photonics Research Center, School of Optoelectronic Engineering, Guilin University of Electronic Technology, Guilin 541004, China

**1. Visualization of features in ASFB**

To verify that the feature transformation in ASFB is consistent with the inversion rules of FTA, we visualized the intermediate feature maps inside ASFB, as shown in Figure S1. The spatial features extracted by SFM are first transformed into the Fourier domain to form multi-channel feature spectra. Subsequently, the network layers in ASFB process the real and imaginary parts of these spectra to generate spectral attention maps. Finally, the attention maps are multiplied with the feature spectra, and the filtered spectra are transformed back into the spatial domain through inverse Fourier transform.

Figure S2 presents representative multi-channel features at different stages of ASFB. Before Fourier transform, the spatial feature maps mainly retain fringe-structure features, and features with clearer fringes correspond to higher absolute response values. After Fourier transform, the spectral responses in most channels are concentrated around the zero-order and ±1^st^ order spectral regions, indicating that the spatial fringe information has been converted into physically interpretable spectral components. In addition, the generated spectral attention weights are mainly distributed around the target spectral region, rather than being uniformly distributed over the entire frequency domain. It should be noted that, unlike conventional FTA, ASFB operates on multi-channel feature spectra instead of directly applying a fixed window to a single input spectrum. Therefore, not every channel-wise attention map focuses on enhancing the +1^st^ order spectral region; a small number of channels may suppress the target region or its neighboring regions, reflecting the ability of the neural network to jointly regulate informative and redundant components in the multi-channel feature space. After multiplication with the attention maps, the filtered spectral components are also mainly concentrated around the +1^st^ order spectral region, while irrelevant spectral components are suppressed. The features obtained after inverse Fourier transform can then preserve the main sample-related spatial structures and are used to reconstruct the phase distribution. These visualizations collectively indicate that the internal inference process of ASFB follows the basic principles of FTA inversion.

**Figure S1.** Inference pipeline and intermediate feature transformation process of ASFB.

**Figure S2.** Complete multi-channel feature maps during the inference process of ASFB, including spatial features, feature spectra, spectral attention maps, filtered spectra, and inverse-transformed spatial features.

**2. Digital holographic interferometry data simulation**

For data simulation, a forward imaging model based on off-axis DHI was constructed. The overall workflow is illustrated in Figure S3(a). First, the three-dimensional refractive index distribution of the fiber sample was generated. The fiber types include multi-core fiber, coaxial fiber, and graded-index multimode fiber. Subsequently, the projection phase at a specific viewing angle was calculated based on the optical path difference model, with the expression given by:

(S1)

where Δ*n* represents the difference between the refractive index of the fiber and that of the surrounding medium, and *λ* is the wavelength of the incident laser. After obtaining the object projection phase, an affine transformation was applied to simulate random tilts and minor jitters of the sample during the rotation process. The relationship between the original coordinates (*x*, *y*) and the transformed coordinates (*x*', *y*') is given by:

$\left[\begin{matrix} x' \\ y' \\ 1 \end{matrix}\right]=\left[\begin{matrix} \cos\beta& -\sin\beta& 0 \\ \sin\beta& \cos\beta& 0 \\ 0 & 0 & 1 \end{matrix}\right]\left[\begin{matrix} 1 & 0 & 0 \\ 0 & 1 & -dy \\ 0 & 0 & 0 \end{matrix}\right]\left[\begin{matrix} x \\ y \\ 1 \end{matrix}\right],$ (S2)

where *β* denotes the clockwise rotation angle of the object around the center point, and *dy* is the random vertical displacement. Subsequently, the projection phase, the aberration term generated by Zernike polynomials, and the spatial carrier frequency term were superimposed and encoded into the interferogram:

(S3)

where *P*_k_ represents the *k*-th Zernike polynomial mode in the Cartesian coordinate system, and *γ*_k_ is the corresponding coefficient. Subsequently, the wrapped phase was constructed as the data label:

(S4)

and the simulated interferogram was generated as the input:

(S5)

where *A*_0_ is the intensity amplitude, taking values between 0.125 and 0.454. *x*_0_ and *y*_0_ are the Gaussian beam offsets in the *x* and *y* directions, respectively, varying from −0.2 to 0.2. *σ* is the beam width control parameter, which ranges from 3 to 5.

Since the simulated interference fringes exhibit sinusoidal structure, they still differ from the degraded fringes recorded in real scenarios. Therefore, a degradation process was further added in the simulation. First, the intensity of the clean fringes was clipped to [0,1] and mapped to the grayscale range of [0,255] to mimic the camera quantization process. Subsequently, we added Gaussian and gamma noise with intensity levels ranging from 0.001 to 0.05 to the clean fringes. The noisy image was then clipped again to the range of [0, 255]. Figure S3(b) presents the noisy interferogram and its corresponding Fourier spectrum. To further simulate the exposure mismatch effect in experimental recording, a linear grayscale transformation was applied to the noisy fringes:

$I_{\deg}(x,y)=s\cdot I_{\text{noisy}}(x,y)+b,$ (S6)

where *s* is a scaling factor controlling the pattern intensity, ranging from 0.8 to 1.2. *b* is an intensity offset ranging from 0 to 2. Larger values of *s* or *b* cause some pixels to be clipped to 255, which results in over-saturation effects, as illustrated in Figure S3(c).

**Figure S3.** Data simulation for DHI. (a) Generation of clean interferogram. (b) Simulated noisy pattern. (c) Simulated overexposure pattern.

**3. Attention map visualization in ASFB**

The spectral attention generated by ASFB contains multiple channel-wise attention maps and therefore cannot be directly displayed as a single image. To visualize the overall spectral attention distribution, the channel-wise attention maps are aggregated according to the response strength of their corresponding complex feature spectra, as illustrated in Figure S4.

Specifically, for the complex feature spectrum $\tilde{F}_{c}$ of the *c*-th channel, its scalar importance weight s*_c_* is calculated as the sum of the spatially averaged absolute responses of the real and imaginary components:

$s_{c}=\frac{1}{HW}\sum_{i=1}^{H} \sum_{j=1}^{W} \left|\mathrm{Re}\left(\tilde{F}_{c}(i,j)\right)\right|+\frac{1}{HW}\sum_{i=1}^{H} \sum_{j=1}^{W} \left|\mathrm{Im}\left(\tilde{F}_{c}(i,j)\right)\right|,$ (S7)

where *H* and *W* denote the height and width of the feature spectrum, respectively. Let **W***_c_* denote the spectral attention map of the *c*-th channel. The final single-channel visualization map **V** is obtained by weighted summation:

$\mathbf{V}=\sum_{c=1}^{C} s_{c}\mathbf{W}_{c},$ (S8)

where *C* is the number of feature channels. Thus, attention maps associated with feature-spectrum channels having stronger real and imaginary responses contribute more substantially to the final visualization.

**Figure S4.** Visualization procedure of the spectral attention map in ASFB. Since the spectral regions corresponding to the zero-order and conjugate components have been masked out by the SM (with attention weights set to zero), only the regions not suppressed by the SM are visualized in this procedure.

**4. Spectral analysis results of phase demodulation on simulated data**

To examine the physical plausibility and accuracy of the spectral attention generated by ASFB, spectral analyses were conducted on three representative simulated cases with different fringe densities, degradation levels, and phase-variation directions, as shown in Figure S5. Figure S5(a) presents the input interferograms and their corresponding Fourier spectra, the +1^st^ order spectrum of the GT, the +1^st^ order spectra corresponding to the phase-demodulation results of FTA and ASFNet, and the filtering-weight distributions of FTA and ASFNet. For Case 1, which contains a relatively clean fringe pattern, the spectral energy is mainly concentrated in the zero-order and ±1^st^ order spectral regions. Both FTA and ASFNet assign relatively high weights around the target +1^st^ order spectrum. In contrast, Case 2 is affected by stronger noise and overexposure, resulting in a more complex spectral distribution and more pronounced secondary spectral components around the +1^st^ order spectrum. Case 3 has a higher fringe density, and the ±1^st^ order spectra are farther away from the zero-order spectrum. Although the spectral characteristics differ substantially among the cases, ASFNet can dynamically adjust the regions on which its attention is concentrated according to the input instance. To visually demonstrate the dynamic sensing capability of ASFB, the local logarithmic spectral-amplitude profiles in Figure S5(b) further reflect the accuracy of ASFNet in sensing the +1^st^ order spectrum. First, the spectral curves corresponding to the ASFNet-demodulated phase are highly consistent with those of the GT. Second, the dynamically generated attention of ASFNet is mainly concentrated in regions with strong responses in the spectrum of the demodulated phase. At locations corresponding to local peaks in the GT spectrum, the attention weights generated by ASFB usually also exhibit local maxima. This correspondence indicates that ASFB can accurately sense the +1^st^ order spectral components and adjust its weight distribution according to the spectral characteristics of the input. In contrast, the weights of FTA are determined by a predefined Hanning window, and the logarithmic spectral-amplitude curves corresponding to its demodulated phase exhibit certain deviations from those of the GT.

**Figure S5.** Spectral analysis of phase demodulation. (a) Representative interferograms with different degradation levels and fringe densities, along with the filter weight distributions generated during the inference processes of FTA and ASFNet, and the corresponding filtered +1^st^ order spectra. (b) Logarithmic spectral amplitude curves along the selected spectral lines L1-L6 and the filter weight curves W1-W6. The black arrows indicate the local spectral peaks and the corresponding local peaks of the attention weights generated by ASFNet.

**5. Quantitative evaluation metrics**

For quantitative comparison, MAE and RMSE were employed to evaluate the numerical deviation between the results and the ground truth, while PSNR and SSIM were used to assess their fidelity. The definitions of each evaluation metric are as follows:

(S9)

(S10)

$\text{PSNR}=10\log_{10}\left(\frac{L^{2}}{MSE}\right),$ (S11)

(S12)

where *W* and *H* are the width and height of the predicted result, respectively. *L* is the upper bound of the pixel value. *μ* and *σ* denote the mean and covariance, respectively. *C*₁ and *C*₂ are set to 0.01 and 0.03, both of which are commonly used empirical parameters.

**6. Generalization testing on experimental interferograms**

Figure S6(a) provides additional reconstruction results on real experimental interferograms to further evaluate the reconstruction stability of ASFNet. These data were acquired at different times and therefore exhibit noticeable differences in overall degradation styles. Compared with FTA and ResUNet, the phase reconstructed by ASFNet shows a smoother background and fewer local artifacts while preserving the main phase structures. The phase profiles in Figure S6(b) further indicate that the ASFNet results are in good agreement with those obtained by well-configured FTA and avoid the obvious spikes observed in the ResUNet results.

**Figure S6.** Additional generalization testing on experimental interferograms. (a) Reconstructed object projection phase under different scenarios. (b) Cross-sectional phase profiles along the white dashed lines in (a).
